# Supplementary material for: Superconductivity onset above 60 K in ambient-pressure nickelate films
Source: Natl Sci Rev. 2026 Mar 10;13(9):nwag151. doi: 10.1093/nsr/nwag151 (PMC13218385; doi:10.1093/nsr/nwag151)
Supplement: nwag151_Supplemental_File [file nwag151_supplemental_file.docx]

Supplementary Data for **Superconductivity onset above 60 K in ambient-pressure nickelate films**

Guangdi Zhou^1,2†^, Heng Wang^1,2†^, Haoliang Huang^1,2†^, Yaqi Chen^1^, Fei Peng^1^, Wei Lv^1^, Zihao Nie^1^, Wei Wang^1^, Qi-Kun Xue^1,2,3*^, Zhuoyu Chen^1,2*^

^1^State Key Laboratory of Quantum Functional Materials, Department of Physics and Guangdong Basic Research Center of Excellence for Quantum Science, Southern University of Science and Technology, Shenzhen 518055, China

^2^Quantum Science Center of Guangdong-Hong Kong-Macao Greater Bay Area, Shenzhen 518045, China

^3^Department of Physics, Tsinghua University, Beijing 100084, China

^†^These authors contributed equally.

^*^E-mail: chenzhuoyu@sustech.edu.cn, xueqk@sustech.edu.cn

**
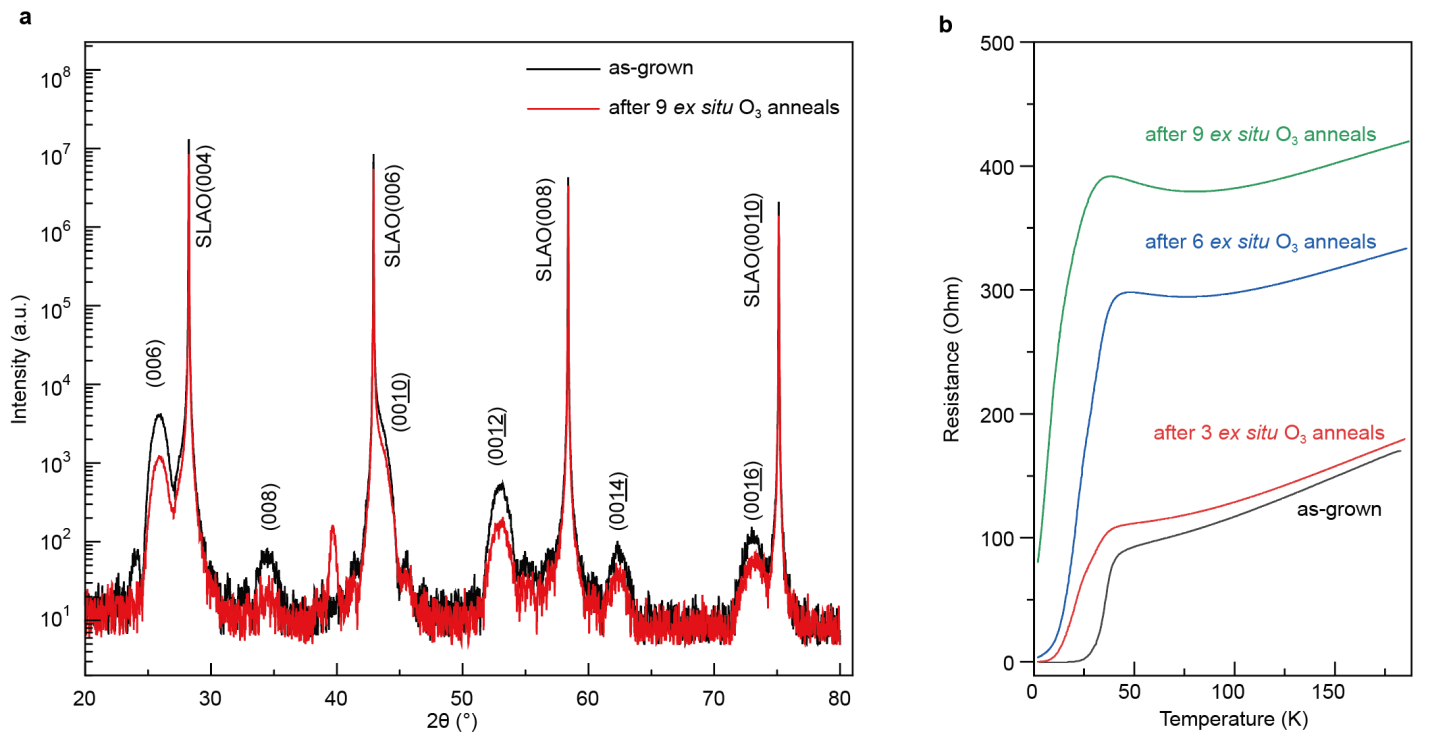
**

**Supplementary Data Figure S1. Structural and transport degradation from repeated *ex situ* post-annealing.** **a,** Out-of-plane XRD θ-2θ scans for an as-grown RP bilayer nickelate film (black curve) compared to the same film after 9 repeated *ex situ* ozone annealing cycles (red curve). The pronounced suppression of the film diffraction peaks demonstrates pronounced structural degradation. **b,** Corresponding resistivity-temperature (*R*-*T*) curves for the same sample. Together, these data display that the *ex situ* annealing process, while necessary to induce superconductivity in the conventional two-step approach, introduces cumulative damage. This implies that even a single anneal, which may not show obvious degradation in XRD, can introduce subtle structural imperfections that limit the film’s crystalline quality and thus the maximum achievable *T*_c_.


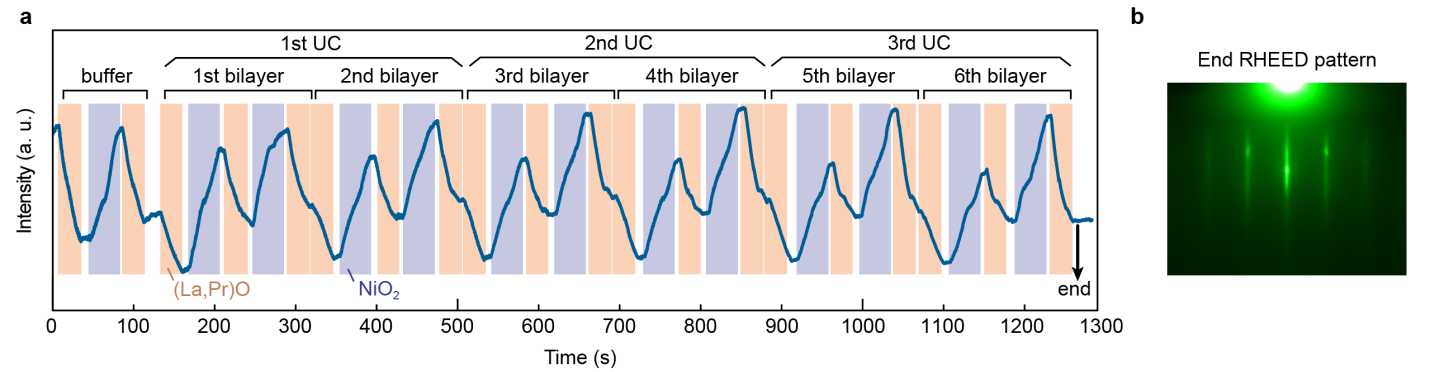


**Supplementary Data Figure S2. GAE growth and reflective high-energy electron diffraction (RHEED) monitoring of a (La,Pr)_3_Ni_2_O_7_ film.** **a,** *In situ* RHEED intensity oscillations recorded during the GAE synthesis of a 3 unit cell (UC) (La,Pr)_3_Ni_2_O_7_ film with a buffer layer. The growth sequence consists of alternating depositions of (La,Pr)O (orange blocks) and NiO_2_ (purple blocks) atomic layers. The persistent oscillations confirm the atomic-layer-by-layer growth mode, allowing for precise control over the stacking sequence and total film thickness. The hierarchy (buffer, 1st UC, etc.) is labeled. **b,** The RHEED pattern at the end of the 3 UC growth. The sharp, streaky pattern indicates a 2D growth mode and confirms the film possesses high crystalline quality and an atomically smooth surface morphology.

**
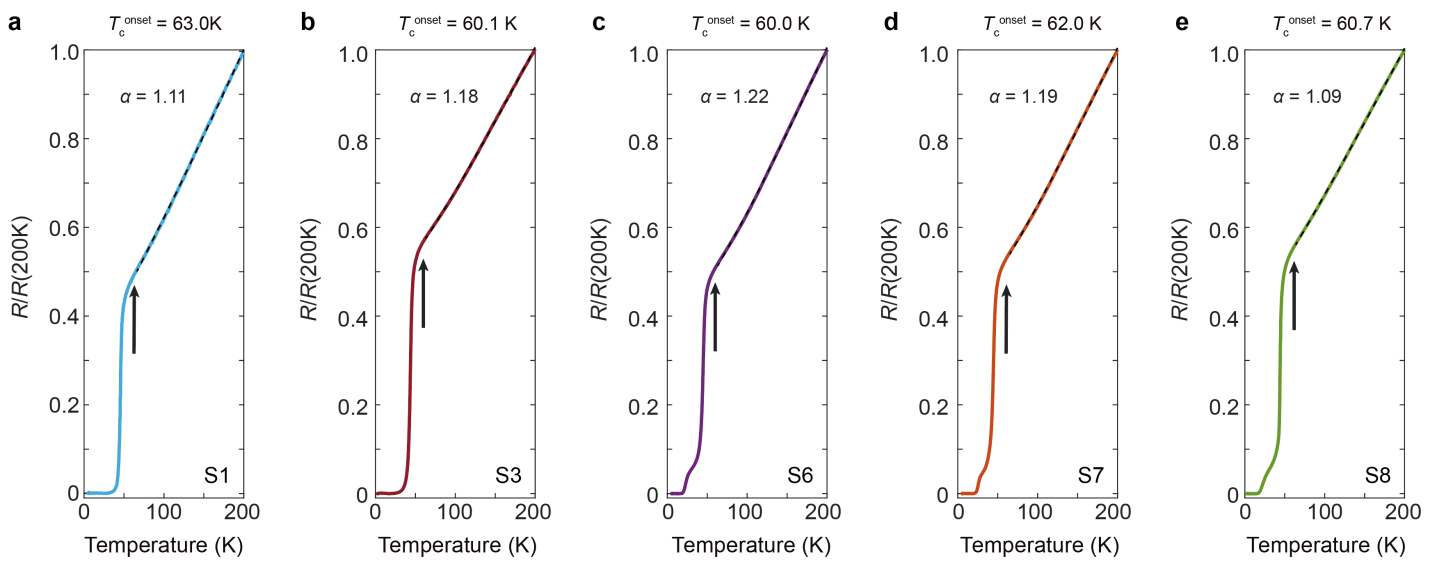
 Supplementary Data Figure S3. Robustness of the strange-metal-like behavior in higher-*T*_c_ samples.** Normalized resistivity-temperature curves for five different (La,Pr)_3_Ni_2_O_7_ samples (S1, S3, S5, S7, S8). Despite variations in the normal-state resistance and the existence of a second step in the superconducting transitions, all samples in this *T*_c_^onset^ regime (ranging from 60.0 K to 63.0 K) consistently exhibit a quasi-linear resistivity (i.e., *α* close to 1). This demonstrates that the strange-metal-like behavior is a robust feature of the 60 K superconducting phase.

**
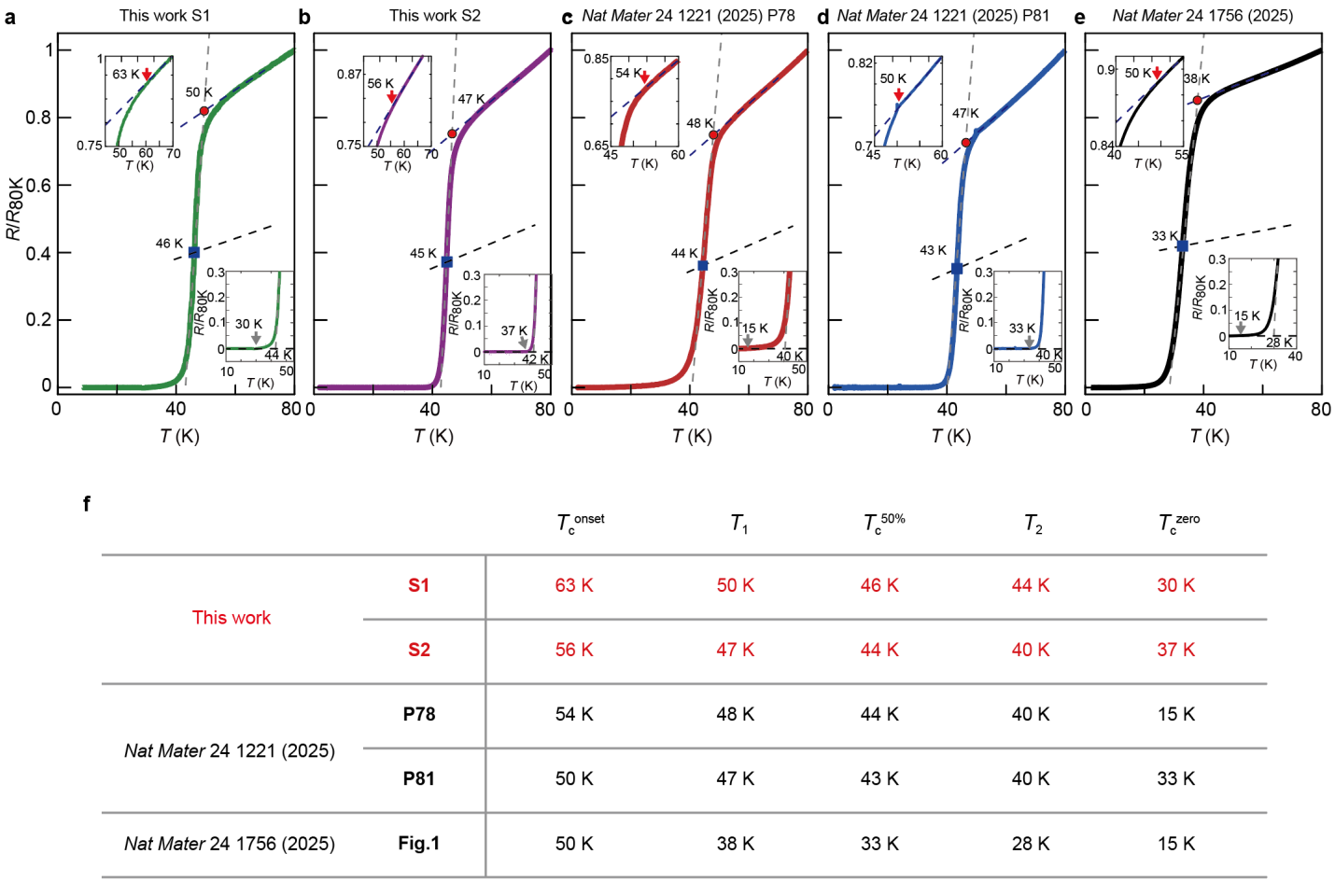
**

**Supplementary Data Figure S4. Detailed definition of transition temperatures and comparison with literature. a–e,** Normalized resistivity-temperature (*R*/*R*_80K_) curves near the superconducting transition for: (a, b) representative films from this work (S1 and S2), (c, d) representative films from *Nat. Mater.* 24, 1221 (2025) (samples P78 and P81), and (e) a representative film from *Nat. Mater.* 24, 1756 (2025). To facilitate a systematic comparison, we define five characteristic temperatures: *T*_c_^onset^ (red arrows in upper insets): The temperature where the resistance deviates from a linear fit to the normal-state data near transition (blue dashed lines). *T*_1_ (red circle): The intersection point between the linear fit of the normal state and the linear fit of the resistive transition edge (gray dashed lines). *T*_c_^50%^ (blue squares): The temperature where the resistance drops to 50% of the value extrapolated from the normal-state linear fit. *T*_2_ (intersections of gray dashed line and black dashed line in lower insets): The intersection point between the linear fit of the transition edge and the zero-resistance axis. *T*_c_^zero^ (gray arrows in lower insets): The temperature where the resistance drops below the measurement noise level. **f,** Summary table comparing the extracted transition temperatures (*T*_c_^onset^, *T*_1_, *T*_c_^50%^, *T*_2_, and *T*_c_^zero^) for the samples shown in a–e.

**
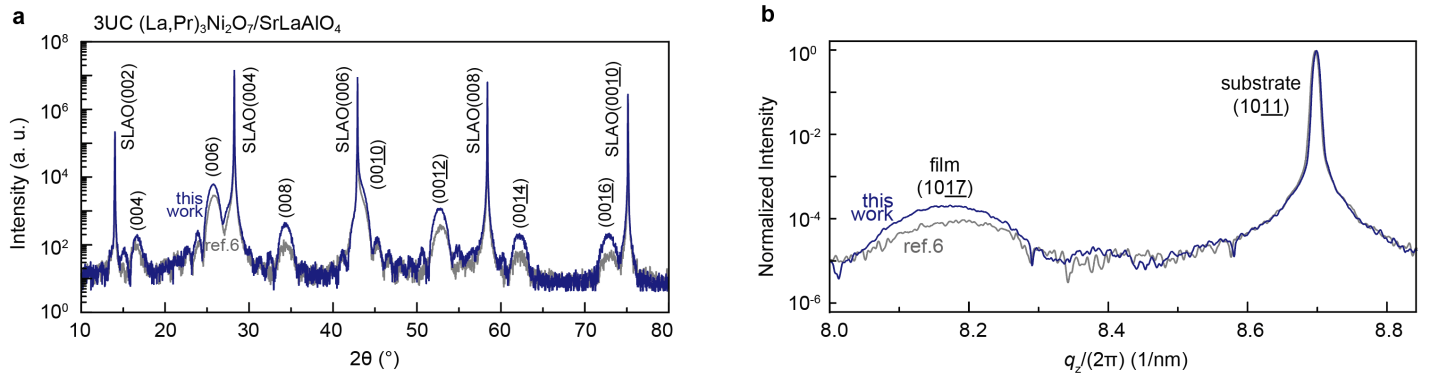
**

**Supplementary Data Figure S5. Comparison of crystalline quality with previous work.** **a,** Comparison of out-of-plane XRD θ-2θ scans for a 3UC (La,Pr)_3_Ni_2_O_7_ film grown in this work (blue curve) and a film from ref. 6 (gray curve). The slight peak position shift is associated with the of chagne of La:Pr ratio (65:35 in this work versus 95:5 in ref. 6). **b,** Comparison of the normalized intensity profiles extracted from RSM scans around the film’s (1017) peak for the same two samples.

**
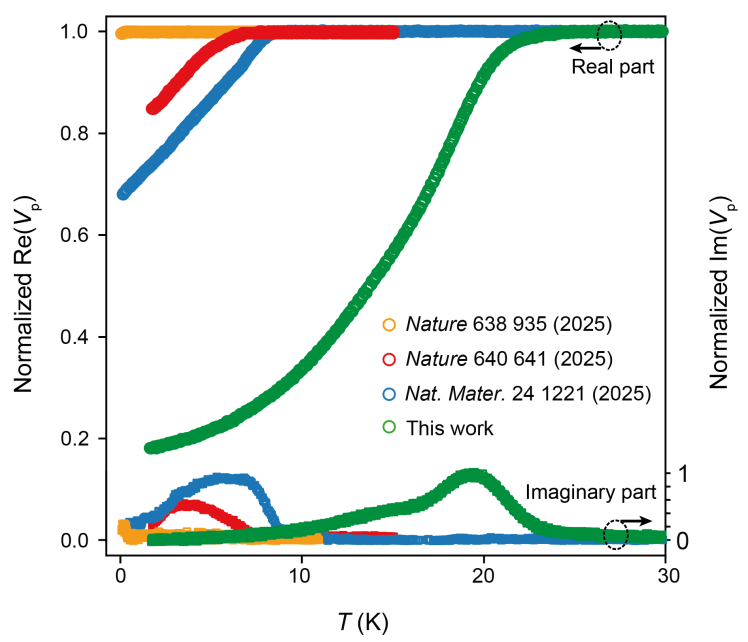
**

**Supplementary Data Figure S6. Comparison of mutual inductance diamagnetic signals.** Temperature dependence of the real part of the pickup coil voltage (normalized) for the (La,Pr) _3_Ni_2_O_7_ film in this work (green) compared with previous reports: *Nature* 638, 935 (2025) (yellow), *Nature* 640, 641 (2025) (red) and *Nat. Mater.* 24, 1221 (2025) (blue).
